# Poisoning emergency visits among children: a 3-year retrospective study in Qatar

**DOI:** 10.1186/s12887-015-0423-7

**Published:** 2015-08-28

**Authors:** Abdelrahman Ahmed, Ashraf Nazmi AlJamal, Mohamed Izham Mohamed Ibrahim, Khalil Salameh, Khalid AlYafei, Samah Abu Zaineh, Fathea Salama S S Adheir

**Affiliations:** Drug Information Unit, Pharmacy Department, Al Wakra Hospital, Al Wakra, Qatar; College of Pharmacy, Qatar University, Al Tarfa, P.O. Box 2713, Doha, Qatar; Paediatric Emergency, Al Wakra Hospital, Al Wakra, Qatar; Pharmacy Department, Women Hospital, Al Wakra, Qatar; Pharmacy Department, Al Wakra Hospital, Al Wakra, Qatar

**Keywords:** Accident & emergency visits, Children, Government hospital, Poisoning, Qatar

## Abstract

**Background:**

Poisoning in toddlers and infants is almost always unintentional due to their exploratory behavior, which is different from adults. The prevalence and background of childhood poisoning in Qatar is still unknown. The aim of this study is to explore the extent of childhood poisoning in Qatar and, specifically, to describe the frequency of poisoning as a cause of Accident & Emergency (A&E) admission, the demographic profile of affected patients, the circumstances leading to exposure, and the specific agents involved in poisoning among children under age 14 in our setting.

**Methods:**

This study was a cross-sectional survey of children up to 14 years old utilizing retrospective data between October 2009 and October 2012. The data were collected from the childhood poisoning case registry and patient medical records at the Accident and Emergency (A&E) Unit of all the Hamad Medical Corporation hospitals. Pharmacists reviewed all the handwritten medical records. Data written on the data collection form were transferred into excel and later into SPSS version 21. The data were analyzed using frequencies and percentages, and a chi-square test was used for categorical variables.

**Results:**

Out of 1179 registered poisoning cases listed in the registry, only 794 cases (67.3 %) were usable and included in the final analysis. A&E admissions for unintentional poisoning for children accounted for 0.22 % of all A&E admissions from 2009 to 12. The majority of poisoning cases happened among children between 1 and 5 years old (*n* = 704, 59.7 %). Cases were more frequent among non-Qatari than Qatari children (39.4 % vs. 28.5 %). Most cases occurred in the living room (28.2 %) and typically took place in the afternoon (29.2 %). Analgesic and antipyretic medicines were the most common agents ingested by children (*n* = 194, 36.9 %), specifically paracetamol (*n* = 140, 26.6 %).

**Conclusions:**

Cases of unintentional poisoning are higher among children aged 1 to 5 years, males and non-Qatari. Most cases occurred in the living room and typically took place in the afternoon. The most common type of poison ingested by children was medicines, i.e., analgesics and antipyretics, specifically paracetamol.

## Background

Poisoning refers to an injury resulting from exposure to an exogenous substance that causes cellular injury or death. Poisons can be inhaled, ingested, injected or absorbed. Poisoning may also be acquired in utero. The exposure may be acute or chronic, and the clinical presentation varies accordingly. The factors determining the severity of poisoning and its outcomes in a child are interrelated. These include the type of poison, the dose, the formulation, the route of exposure, the age of the child, the presence of other poisons, the state of nutrition of the child, and the presence of other diseases or injuries [1, 2]. According to the World Health Organization (WHO) and the United Nations Children’s Fund (UNICEF), poisoning in childhood is common because children are curious and explore their world with all their senses, including taste [1]. Most of the time they are at home, and the home and its environments can be an unsafe place in which poisonous substances are unintentionally ingested.

Poisoning is a significant global public health problem. The extent of the problem is different from 1 country to the other. In 16 high-income and middle-income countries, poisoning is the fourth biggest cause of unintentional injury after road traffic injuries, fires and drowning [1]. Further, according to data from the WHO, an estimated 350 000 people died from unintentional poisoning in 2002. In 2004, acute poisoning caused more than 45 000 deaths in children and youth less than 20 years of age. It is the second most common cause of injury resulting in the hospitalization of children under the age of five years [1].

While accidental poisoning occurs most often in the previously mentioned age group, less than 1 % of poisoning cases in children are serious. The most common agents involved are over-the-counter (OTC) medications, prescription medications, household products, paraffin/kerosene, pesticides, poisonous plants and animal or insect bites [2]. Identifying those at risk at an early stage remains a challenge. Education of the general public and the provision of childproof containers for household chemicals and medicines play a key role in prevention [3]. The time interval between the exposure to poison and the appearance of clinical symptoms presents an important window of opportunity. During this period, it is important to minimize absorption by removing or neutralizing the poison (in the case of ingestion) or by administering agents that prevent damage to the organs.

According to a report from the WHO [1], fatal poisoning rates in low-income and middle-income countries are four times that of high-income countries. Africa and low-income and middle-income countries in Europe and the Western Pacific Regions have the highest rates. Common poisoning agents in high-income countries include pharmaceuticals, household products (e.g., bleach and cleaning agents), pesticides, poisonous plants and bites from insects and animals. Common poisoning agents in low-income and middle-income countries are fuels such as paraffin and kerosene, pharmaceuticals and cleaning agents. In some countries, poisoning death rates are highest in children less than one year old, while non-fatal poisonings appear to be more common among children aged 1 to 4 years. Studies from both low-income and high-income countries suggest that poisonings and their management are costly.

A lack of adult supervision at home leads to some 500 cases of poisoning among children in Qatar every year, caused by the consumption of substances such as medicines, cosmetics or chemicals [4]. According to this report, approximately 40,000 children are injured yearly due to several reasons including poisoning and are rushed to emergency units for medical attention; alarmingly, 85 % of these events occur at home. As a result, there is an increasing pressure on the emergency facilities of the Hamad Medical Corporation (HMC) to meet the demands for hospital care. These accidents are preventable because the main reasons for such incidents are a lack of knowledge, a lack of awareness and a lack of adult supervision. Further, it is reported that most poisoning events involve medicines, household products and cosmetics, but no study has verified this claim. If we are to make progress in reducing childhood injury from pharmaceutical and chemical poisoning, we need to better understand the epidemic. A study assessing the medications that are the most consequential—those that contribute the most emergency department (ED) visits, hospitalization, and harm, is needed.

Although it is a global phenomenon, poisoning among children has unique epidemiological attributes depending on socio-economic demographics. Therefore, there is a need for a study in this part of the world to understand the unique epidemiological characteristics in Qatar to effectively address the problem of childhood poisoning.

The aim of this study is to explore the extent of childhood poisoning in Qatar. Specifically, the primary objectives of this study are to describe the frequency of poisoning as a cause of A&E admission, the demographic profile of affected patients, the circumstances leading to exposure, and the specific agents involved in poisoning among children less than 14 years old in our setting.

## Methods

### Study design

This research is a cross-sectional descriptive study utilizing retrospective data. The study was led by the drug information pharmacists at Al-Wakra Hospital, Al-Wakra, Qatar.

### Ethics consideration

This study was conducted according to the government regulations and institutional research policies and procedures in Qatar. The study protocol was approved by the Hamad Medical Ethics Committee (Research Proposal No: 12253/10). The need for subject informed consent was waived by Hamad Medical Ethics Committee.

### Study location and period

The data and information from October 2009 to October 2012 were collected from the A&E Unit of all hospitals managed under the Hamad Medical Corporation (HMC).

The Hamad Medical Corporation is the principal public healthcare provider for the State of Qatar. In addition to three general hospitals (Al Khor Hospital, Al Wakra Hospital and The Cuban Hospital), HMC also manages five specialist hospitals (Hamad General Hospital, Women Hospital, Women’s Hospital, the National Center for Cancer Care and Research and Heart Hospital) that care for patients with the most prevalent conditions including cancer and heart conditions, and they provide specialist treatment and rehabilitation for women and children. HMC also operates the national Ambulance Service and a home healthcare service [5].

### Population and sampling

The target population for the study was all children aged 14 years or younger presenting with poisoning at any HMC A&E during the study period. The information about the study population was gathered from the childhood poisoning case registry in the A&E unit. The study excluded intentional poisoning cases if mentioned in the case registry; these cases are few due to cultural practices and beliefs. This study only examined unintentional poisoning cases.

### Data collection procedure

The data were collected from hospital medical records. Pharmacists reviewed all the handwritten medical records. All of the required information was extracted and transferred onto a data collection form. The data collection form was developed and pretested before it was used. The collected information included patient demographic profiles (i.e., age, gender and nationality), the type of poison, the time of arrival at the hospital after exposure, the time of exposure, the season when the event occurred, the length of the hospital stay, the storage condition of the poison, the medical intervention and the outcome of the unintentional poisoning.

### Data management and analysis

Data recorded on the data collection form were transferred into an excel program, and then later into SPSS version 21. The data were analyzed descriptively using frequencies and percentages. A chi-square test was used for categorical data with an alpha level of 0.05.

## Results

Of the 1179 registered poisoning cases listed in the registry, only 794 cases (67.3 %) were usable and included in the final analysis. The rest of the records were excluded due to the unavailability of medical records or missing data. A&E admissions for unintentional poisoning for children accounted for 0.22 % of all A&E admissions from 2009 to 2012.

The following Table presents the number of emergency cases during the study period (Table 1).

**Table 1:** Total number of registered cases from 2009 to 2012

Diagnosis in general	Frequency (%)
Respiratory disorder	470402 (86.4)
Common cold	25750 (4.7)
Gastrointestinal disorder	17889 (3.3)
Skin disorders	16019 (2.9)
Eye and Ear disorder	6717 (1.2)
Trauma	5599 (1.0)
Poisoning	1179 (0.3)
Neurological disorder	794 (0.2)
Total	544,349

The total number of A&E visits (for children aged 14 and younger) that occurred during the study period was 544,349. Based on data availability, the statistics indicated that there were 259,584 (54.2 %) male cases compared to 219,020 (45.8 %) female cases; and Qatari children comprised 163,963 cases (33.9 %) compared to 320,240 cases (66.1 %) among non-Qatari children.

Table 2 illustrates the association between the child age groups and gender and nationality. No significant association was found.

**Table 2:** The association between age groups of admitted children with gender and nationality

	Age group				
Characteristics	(1–5)	(6–9)	(10–14)	Total	*p* value
	*n* (%)	*n* (%)	*n* (%)		
Gender					
Male:	385 (88.7)	40 (9.2)	9 (2.1)	434	0.95 (NS)
Female:	320 (89.1)	31 (8.6)	8 (2.3)	359	
Nationality					
Qatari:	298 (88.7)	30 (8.9)	8 (2.4)	336	0.99 (NS)
Non-Qatari:	406 (88.6)	41 (9.0)	11 (2.4)	458	

The findings in Table 3 demonstrate that for most of the poisoning cases, the time of exposure before visiting the A&E department is less than 1 h. (54.2 %). Most of the cases happened in the living room (28.2 %), followed by in the kitchen (15.5 %). Poisoning cases usually occurred in the afternoon (29.2 %), followed by the evening (24.3 %). In terms of identifying whether poisoning cases are seasonal, cases occurred more frequently between June and August, during which the number of cases was only slightly higher than other periods. For the majority of cases, the patients were hospitalized between 1–4.9 h (35.2 %) and 67.0 % of the patients were discharged. Further findings showed that the most common route of exposure is oral (66.7 %, *n* = 786). The most common type of poisons experienced by children was medicine (72.6 %), and the most common type of non-medicine related poisons was household items (14.9 %). The non-medicine types of poisons were Clorox (51 cases), mice poisoning (30 cases), Dettol (28 cases), detergent (10 cases), acetone (7 cases), boric acid (7 cases), and other agents/substances.

**Table 3:** Poison-related information and type of poisoning management

Items		Frequency (%)
Time of exposure before visiting the A&E Department	Less than 1	639 (80.5)
	1 to less than 2	154 (19.4)
	2 to 3	1 (0.1)
	More than 3	Nil
Poisoning place	Living room	333 (42.2)
	Kitchen	183 (23.1)
	School	1 (0.1)
	Others	273 (34.6)
Time of exposure	Morning	157 (20.0)
	Afternoon	344 (43.7)
	Evening	286 (35.3)
Months (Seasons) of exposure	December-February (Winter)	177 (22.4)
	March-May (Spring)	204 (25.8)
	June-August (Summer)	211 (26.7)
	September-November (Fall)	199 (25.1)
Type of poisons	Medicines	530 (72.6)
	Non-medicine	200 (27.4)
Non-medication poisons (*n* = 200)	Household items	176 (88.0)
	Insecticide	24 (12.0)
Type of management	Chelating agent i.e., charcoal	506 (64.2)
	Emesis	Nil
	Antidotes	Nil
	Acetylcystine	5 (0.6)
	Observation	278 (35.2)
Length of hospital stay	Less than 1 h.	49 (6.2)
	1 to 4.9 h	415 (52.3)
	5 to 9.9 h	282 (35.6)
	More or equal to 10 h	47 (5.9)
Outcome of the management	Patient was discharged	790 (99.5)
	Patient was referred	4 (0.5)

Note: Total frequency is not equal to 794 due missing data

Table 3 also presents the type of management provided to the patients. In the majority of cases, the children were managed using a chelating agent i.e., charcoal (42.9 %), in 23.6 % of the cases, the children were put under observation and 67 % of the patients were discharged from the A&E. The rest were admitted for inpatient hospitalization.

The information on the type of medicines commonly ingested by children is indicated in Table 4 below. Analgesic and antipyretic medicines are the most common agents ingested by children (*n* = 194, 36.9 %), followed by antihypertensive agents (*n* = 55, 10.5 %), then antihistamines (*n* = 42, 8.0 %); paracetamol is the most common medicine (*n* = 140, 26.6 %) ingested by children. The source of poison, in order of frequency, was from the original container (*n* = 609, 51.7 %), a food container (*n* = 2, 0.2 %), a soda bottle (*n* = 1, 0.1 %), and a juice bottle (*n* = 1, 0.1 %). The poisons were obtained from the refrigerator in 13.3 % (*n* = 157) of cases (Table 3).

**Table 4:** The association between age groups of children and classes of medicines ingested

	Age group			
Medicines class	(1–5)	(6–9)	(10–14)	Total
	*n* (%)	*n* (%)	*n* (%)	
Analgesic and antipyretic (*n* = 194)	148 (76.3)	37 (19.1)	9 (4.6)	194
Antihypertensive (*n* = 55)	53 (96.4)	2 (3.6)	0 (0)	55
Antihistamines & cough mixture (*n* = 42)	35 (83.3)	6 (14.3)	1 (2.4)	42
Vitamins and minerals (*n* = 42)	38 (90.5)	3 (7.1)	1 (2.4)	42
Antihyperglycemic (*n* = 23)	21 (91.3)	2 (8.7)	0 (0)	23
Anticonvulsant (*n* = 21)	17 (80.9)	2 (9.5)	2 (9.5)	21
Hormones (*n* = 24)	20 (83.3)	3 (12.5)	1 (4.2)	24
Antibiotics (*n* = 6)	6 (100)	0 (0)	0 (0)	6
Misc. (*n* = 123)	115 (93.5)	8 (6.5)	0 (0)	123

## Discussion

This study aims to explore the extent of childhood poisoning in Qatar. Specifically, the primary objectives of this study are to describe the frequency of poisoning as a cause of A&E admission, the demographic profile of affected patients, the circumstances leading to exposure, and the specific agents involved in poisoning among children younger than 14 years old in our setting.

According to McGregor et al. [6], Poison Control Centers in the United States received more than 2.4 million reports of toxin exposures in 2003. Most exposures involved oral ingestion and occurred in the home, and more than 80 % were unintentional. Children younger than six years accounted for 51 % of the exposures. Of these, 38 % involved children three years of age or younger. Most ingestions involved nontoxic substances and were managed at home [6]. A report by the WHO indicated that there are strong associations between injuries and a child’s age, the developmental stage of a child, how he/she interacts with the world and activities undertaken [1]. The results for Qatar are somewhat similar to those of other countries: the majority of poisoning cases occurred among children between 1 and 5 years old, and male patients had slightly higher rates of poisoning than females. A study of Victorian public hospitalization in Australia reported that hospital admissions for injury, i.e., unintentional poisoning for children less than 5 years old, accounted for 16.8 % of all hospital admissions [7]. The hospitalization rates and frequencies were higher for males compared to females; and mostly occurred at ages 1 and 2. The NSU Briefing on Childhood Poisoning in Australia indicated that an estimated 14,339 young children were hospitalized as a result of poisoning from 1999–2000 to 2003–2004 [8]. The rates of hospitalized poisoning injury were highest among patients two years of age (males = 366 and females = 338 poisoning admissions per 100,000 population). Studies by Lam [2], Reith et al. [9] and Morrison et al. [10] indicated that children 0-4 years of age were most frequently hospitalized due to poisoning [2, 9, 10].

According to Meyer et al. [11], unintentional household poisoning in Germany always occurred in toddlers and infants due to their behaviors, which supports our research results [11]. Although a significant reduction of poison cases in advanced countries has been achieved, children are still exposed to toxic agents. The products most accessible to children, as seen from our research and compared to other studies, are primarily medication and secondarily cleaning products and cosmetics. This exposure reflects the availability and accessibility of the products due to the lack of safe storage devices and product disposal practices. Cleaning products should be kept or stored on high shelves. In addition, there should be increased awareness among family members regarding this issue. The relevant authority, such as the Qatar Ministry of Health and municipal councils in collaboration with consumer groups, should assess the effectiveness of child resistant product packaging for household items.

A study called the CHIRPP (the Canadian Hospital Injury Reporting and Prevention Program) conducted in an accident and emergency department in the Sick Child Hospital in Canada reflects that injuries commonly occur in a child’s own home, particularly at the age of 0-4 years [12]. The majority of such cases presented with the ingestion of a foreign body. The CHIRPP is a valuable source of information on patterns of childhood injury that may be used to develop, implement and evaluate child injury prevention activity. Slightly over half of the cases were boys, and seasonal variation was observed with most cases occurring during vacation time and at home.

This study found that in most of the poisoning cases, the time of exposure before visiting the A&E department is less than 1 h., and most of the cases happened in the living room, followed by the kitchen, both of which are in the home. There is no clear difference between months or seasons within a year. The reports by Hoy et al. [7], Cripps and Steel [8] and Morrison et al. [10] also indicated that the most common place for the poisoning of younger children in Australia was in the home; they also mentioned adjacent grounds, but this was not reported in our study [7, 8, 10].

In the majority of cases, the children were managed using a chelating agent. The majority of patients were hospitalized for between 1–4.9 h and were then discharged. Hoy et al. [7] reported that 97 % of admissions due to poisoning are shorter than three days’ duration [7].

Due to the nature and environment of the country, non-pharmaceutical poisoning admissions in children from noxious bites from arthropods such as spiders, bees and wasps were not observed, in contrast to the cases reported in countries such as India [13]. The poisoning cases observed in Qatar thus differ from poisoning events in other countries.

Seventy three per cent of poisoning hospitalizations in an Australian study were due to the ingestion of drugs, medications, or biological substances [7]. The remainder were mostly due to exposure to domestic chemicals. In our study, analgesic and antipyretic medicines are the most common agents ingested by the children, and paracetamol is the most common medicine ingested by children. The most common type of non-medicine related poisons was household items and the source of poison was from the original container. Cripps and Steel [8] and Reith et al. [9] also reported that paracetamol was the most common pharmaceutical poisoning diagnosed in one and two year old Australian children [8, 9]. Thus, paracetamol is commonly involved in childhood poisoning. Lam [2] also reported that analgesics are the most frequently associated medication involved in childhood poisoning [2]. A study by Bond et al. [14] indicated that children’s self-exposure to prescription products dominated the health care impact of the visits and the rate of admissions, and it caused significant injuries [14]. The greatest resource use and morbidity followed self-ingestion of prescription products, particularly opioids, sedative-hypnotics, and cardiovascular agents. Unlike Qatar and other countries, kerosene and snakebites were the most common agents in India, where kerosene has remained the single largest contributor to childhood poisoning [13].

The studies described above show that the issue of medication poisoning in children is still a concern and is not improving. Past preventive efforts have proved to be inadequate. More children are exposed, seen in an A&E, admitted, and injured each year. According to Budnitz et al. (2012) [3], despite childproof caps and safety warnings, the number of accidental drug poisonings among young children has increased over the years. This is because prescription drug use by both adults and children is on the rise, and there are simply more bottles of pills in the home that can potentially be accessed by curious children [3, 15]. In other studies, the greatest increases are observed from prescription pharmaceuticals, particularly opioid analgesics, sedative-hypnotics, and cardiovascular medications. In our study, such cases are still few. New efforts must be directed at these important conditions. Educational efforts are important but are unlikely to achieve significant improvements alone. Education interventions should the readdress home storage of all medications, the repackaging of medications, particularly chronic medications, and the fact that older siblings may not be as careful as parents when opening containers or taking medications, in addition to targeting nannies and other caretakers. A study conducted by the Pittsburgh Poison Centre concluded that “a large percentage of the adult population are potential poisoning victims due to their inability to read and comprehend label instructions” [16]. Storage devices and child-resistant closures may need improvement. Additionally, mechanical barriers to ingestion such as blister packs may be required for more substances.

It is clearly that children between the ages of 1–5 years old are the most vulnerable to poisoning. Further, this study also indicated that poisoning from household items and insecticides occurred only among children between the ages of 1–5 years old. The results of this research have multiple applications that may help in reducing the level of unintentional poisoning incidents and in better utilizing internal hospital resources for other areas of concern by: (1) developing public awareness programs and increasing the level of community awareness about the main causes of unintentional poisoning; (2) providing a key for eliminating the common causes of unintentional poisoning cases in future studies; (3) preventing potential children mortality due to unintentional poisoning; (4) enhancing emergency response plans to reduce exposure time to poisoning based on the root cause identified; and (5) enhancing community knowledge on emergency response actions in the event of unintentional poisonings.

### Study strengths and limitations

This was the first study in Qatar and in public hospitals. This study included all hospitals under the HMC. The findings of this study, which were based on a large sample size, will be used to design an intervention study focusing on educational strategies. However, this study suffers several limitations including missing data and the unavailability of some medical records. In addition, the pharmacists experienced difficulties when extracting information from the medical records due to the illegible handwriting of the physicians. The authors did not included a review by a doctor of all illegible medical records.

This study is a retrospective review of hospital records, and therefore subject to several additional limitations that should be noted explicitly. For example, not all cases of poisoning in childhood in Qatar are brought to an A&E, and this may introduce a systematic error. Further, not all cases of poisoning may have been identified as such in the records. The authors could not confirm whether the included cases were identified from admission diagnoses, discharge (final diagnoses), or both. Some cases may have been missed or misclassified by clinicians. This study was also not able to determine the proportion of cases that presented to various healthcare institutions under the HMC organization.

### Recommendations

There are several specific and general suggestions related to practice, policy and future research that various stakeholders should further consideration:

i. The parameters related to poisoning cases in the A&E unit should be properly and completely documented;
ii. Hospitals should establish a database for poisoning cases and information should be captured once parents or children arrive at the A&E department;
iii. It is important to introduce new regulations to cover emergency substances such as dietary supplements, herbal preparations, and traditional remedies;
iv. Social disparity issues must be addressed and included in prevention programs because the poisoning of children is related to the social and economic status of the children;
v. A study of the impact of education on the prevalence and incidence of childhood poisoning is needed;
vi. The Qatar National Poison Prevention & Control Center (Q-NPPCC) should be set up and with a toll-free number 24 h a day and 7 days a week;
vii. Trained doctors, pharmacists and nurses in clinical toxicology are needed to develop hospital poison teams; and
viii. Clinical toxicology should be directed toward the further development of clinic and basic science research.

## Conclusions

Unintentional poisoning cases were higher among non-Qatari male children aged 1 to 5 years old. Most of the cases occurred in the living room. Poisoning cases usually occurred in the afternoon and evening. The most common type of poison experienced by children was medicine, and exposure was commonly oral. Analgesics and antipyretics were the most common class of medicines ingested by children in Qatar. Paracetamol was the most common medicine ingested by children.
